# Safety and Efficacy of Glucomannan for Weight Loss in Overweight and Moderately Obese Adults

**DOI:** 10.1155/2013/610908

**Published:** 2013-12-30

**Authors:** Joyce K. Keithley, Barbara Swanson, Susan L. Mikolaitis, Mark DeMeo, Janice M. Zeller, Lou Fogg, Jehan Adamji

**Affiliations:** ^1^Rush University College of Nursing, 600 S. Paulina Street, Suite 1080, Chicago, IL 60612, USA; ^2^Rush University Medical Center, 1725 W. Harrison Street, Chicago, IL 60612, USA; ^3^Section of Gastroenterology and Nutrition, Rush University Medical Center, 1725 W. Harrison Street, Chicago, IL 60612, USA; ^4^North Park University School of Nursing, 3225 W. Foster Avenue, Chicago, IL 60625, USA; ^5^Community, Systems, and Mental Health Nursing, Rush University College of Nursing, 600 S. Paulina Street, Suite 1080, Chicago, IL 60612, USA; ^6^Faculty Practice, Rush University College of Nursing, 600 S. Paulina Street, Suite 1080, Chicago, IL 60612, USA

## Abstract

*Background*. Few safe and effective dietary supplements are available to promote weight loss. We evaluated the safety and efficacy of glucomannan, a water-soluble fiber supplement, for achieving weight loss in overweight and moderately obese individuals consuming self-selected diets. *Methods*. Participants were randomly assigned to take 1.33 grams of glucomannan or identically looking placebo capsules with 236.6 mL (8 ounces) of water one hour before breakfast, lunch, and dinner for 8 weeks. The primary efficacy outcome was change in body weight after 8 weeks. Other efficacy outcomes were changes in body composition, hunger/fullness, and lipid and glucose concentrations. Safety outcomes included gastrointestinal symptoms/tolerance and serum liver enzymes and creatinine levels. *Results*. A total of 53 participants (18–65 years of age; BMI 25–35 kg/m^2^) were enrolled and randomized. The two groups did not differ with respect to baseline characteristics and compliance with the study supplement. At 8 weeks, there was no significant difference between the glucomannan and placebo groups in amount of weight loss (−.40 ± .06 and −.43 ± .07, resp.) or other efficacy outcomes or in any of the safety outcomes. *Conclusions*. Glucomannan supplements administered over 8 weeks were well tolerated but did not promote weight loss or significantly alter body composition, hunger/fullness, or lipid and glucose parameters. This trial is registered with NCT00613600.

## 1. Introduction

Overweight and obesity are exceedingly difficult to reverse. Despite the widespread use of conventional management strategies—low-calorie diets, physical activity, behavioral interventions, and pharmacological agents—the prevalence of overweight and obesity continues to rise in the US. An estimated 65% of all US adults were either overweight or obese during 2007-2008 [1]. Overweight and obesity increase the risk for comorbidities such as diabetes and atherosclerosis and are associated with reduced quality of life and life expectancy [2, 3]. Clearly, alternative approaches are needed. One potentially promising alternative approach is glucomannan, a dietary supplement widely promoted and used for its weight loss properties. Despite its widespread use, the safety and efficacy of glucomannan have not been adequately studied.

**Figure 1:**
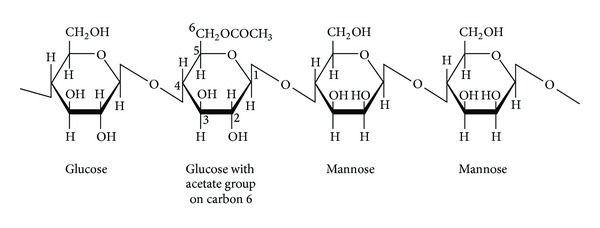
Structure of a segment of glucomannan with repeating glucose and mannose units.

Glucomannan is a water-soluble, fermentable dietary fiber extracted from the tuber or root of the elephant yam, also known as konjac (*Amorphophallus konjac* or *Amorphophallus rivieri*). Glucomannan consists of a polysaccharide chain of beta-D-glucose and beta-D-mannose with attached acetyl groups in a molar ratio of 1 : 1.6 with beta 1–4 linkages (see Figure 1) [4–6]. Because human salivary and pancreatic amylase cannot split beta 1, 4 linkages, glucomannan passes relatively unchanged into the colon, where it is highly fermented by colonic bacteria. It has a high molecular weight (average: 1,000,000 Daltons) and can absorb up to 50 times its weight in water, making it one of the most viscous dietary fibers known [6]. Therefore, glucomannan is taken in smaller doses than other types of fiber supplements.

The mechanisms that mediate the weight reduction effects of glucomannan are thought to be similar to those of other water-soluble, fermentable fibers. With its low energy density and bulking properties, glucomannan seems to promote weight loss by displacing the energy of other nutrients and producing satiety and satiation as it absorbs water and expands in the gastrointestinal tract. In addition, glucomannan seems to reduce total cholesterol and low-density lipoprotein (LDL) cholesterol levels by stimulating fecal excretion of cholesterol and bile acids and decreasing intestinal absorption of cholesterol [7–9]. Also, glucomannan may improve glycemic parameters by inhibiting appetite and slowing intestinal absorption due to increased viscosity [10–13]. Glucomannan is generally well tolerated and has a favorable safety profile.

Glucomannan has been associated with reductions in body weight and plasma lipid and glucose levels in adults in a few clinical trials [14–19]. But these trials have been limited by weak designs, small sample sizes, heterogeneous diagnoses, variable formulations and dosages of glucomannan, and short duration of follow-up [20]. In contrast to these studies, we used a randomized, double-blind, placebo-controlled design to evaluate the safety and efficacy of 3.99 g/day of glucomannan capsules in a sample of 53 healthy overweight and moderately obese adults consuming self-selected diets and maintaining usual physical activity levels during an 8-week study period.

## 2. Materials and Methods

### 2.1. Study Population

Men and women were recruited from a clinic within an urban academic medical center, located in a Health Resources and Services Administration (HRSA) designated medically underserved area. Individuals were eligible for inclusion in the study if they were between the ages of 18 and 65 years and had body mass index (BMI) ≥25 and ≤35 at study entry. Individuals were not eligible if they were currently using fiber supplements or had intolerance to fiber supplements, had untreated/unstable metabolic conditions known to influence weight status (e.g., hypothyroidism, type 2 diabetes mellitus), had gastrointestinal disorders that might cause complications or influence motility or satiety (e.g., diverticulitis, inflammatory bowel disease, irritable bowel syndrome, intestinal narrowing or obstruction, and difficulty swallowing), were using medications or complementary and alternative medicine (CAM) therapies that might affect weight or food absorption (e.g., diuretics, glucocorticoids, anorexigenic agents, Orlistat, acupuncture, and Hoodia), had an eating disorder, or were participating in a weight loss program. Other exclusion criteria were stage II hypertension (≥160/100 mmHg) or dyslipidemia (fasting LDL cholesterol ≥ 160 mg/dL; total cholesterol ≥ 240 mg/dL; triglycerides > 200 mg/dL; HDL ≤ 40 mg/dL), fasting serum glucose > 126 mg/dL, renal or liver disease, history of depression, abuse of illicit drugs or alcohol, use of cigarettes, or pregnant, less than 6 months postpartum, or lactating.

Based on a previous placebo-controlled trial of glucomannan [18], we planned to recruit 50 participants and follow them up for 8 weeks to have 80% power to adequately detect changes in weight and other metabolic variables.

### 2.2. Study Design

Eligible individuals who consented to participate in the study were randomly assigned to receive capsules containing glucomannan or a matching placebo filled with inactive microcrystalline cellulose. A random number generator was used to create a randomization sequence; boxes containing each participant's supply of capsules were packaged according to this sequence. Both the glucomannan and placebo capsules were prepared by an external pharmacy, which had no other role in the study. To ensure that the glucomannan supplement used during the study period met quality control standards, it was purchased from the same lot, and a sample was submitted to ConsumerLab.com for compositional and purity analyses, which indicated appropriate composition and purity. Neither the participants nor the investigators were aware of the treatment assignments.

Participants were instructed to take two 666 mg (1.33 g) glucomannan or placebo capsules with 236.6 mL (8 oz.) of water one hour before breakfast, lunch, and dinner for 8 weeks (for a total of 3.99 g/day). They were also encouraged to maintain their current dietary intake and physical activity levels. Study participants returned at 2 weeks and 8 weeks to return any unused study supplement or placebo from the previous visit, receive a new supply of the study supplement or placebo for the remaining 6 weeks, report on side effects, and have blood drawn.

All data were collected by study research personnel and uploaded to TeleForm (electronic scanning) database by a research assistant. The study was approved by the site institutional review board. All participants provided written informed consent before enrollment. An independent data and safety monitoring committee monitored the trial and reviewed the interim results.

### 2.3. Primary Outcome

The primary efficacy outcome was weight loss from baseline to 2 weeks and 8 weeks after randomization. Body weight was measured to the nearest 1/10 kg using a calibrated electronic scale, with participants wearing light clothing without shoes [21].

### 2.4. Secondary Outcomes

Secondary efficacy outcomes included changes in body composition (waist/hip circumference, body fat, and fat-free mass), hunger and fullness, and fasting lipids and blood glucose parameters. Waist and hip circumference were determined using standardized procedures [22] and body fat and fat-free mass were measured using Tanita Ultimate Scale (Tanita Corp., Tokyo, Japan). Subjective sensations of hunger and fullness were assessed using standardized 100 mm visual analog scales (VAS) [23]. The hunger scale was anchored by the words, “Not at all hungry” and “Extremely hungry” and the fullness scale was anchored by “Not at all full” and “Extremely full.” Participants were asked to make a vertical mark across the line corresponding to their feelings during the past four hours on the day of their scheduled clinic visit (total = three days during the 8-week study period). To score the scales, the distance in mm from 0 for each scale was measured with a ruler. Fasting peripheral venous blood specimens were obtained for glucose and lipid levels. A standard lipid panel was used to quantify triglycerides, total cholesterol, and HDL cholesterol; LDL cholesterol levels were calculated using the Friedewald equation.

Key safety outcomes were gastrointestinal symptoms and tolerability and laboratory assessment of liver and renal function. Gastrointestinal symptoms and tolerance were determined by asking participants about difficulty swallowing, abdominal distention, diarrhea, belching, and other gastrointestinal-related symptoms using standard methods of nondirected questioning, including when symptoms started and whether they were thought to be related to the study supplement. Liver enzymes were considered elevated with an aspartate aminotransferase level >275 u/L and/or an alanine transferase >250 u/L; for serum creatinine, a level > 4.5 mg/dL was considered elevated.

Other measures included dietary intake, physical activity, supplement compliance, and credibility/expectancy perceptions of the study treatment. To assess for changes over the 8-week study period, dietary intake was measured using 3-day food records completed at baseline, 2 weeks, and 8 weeks and analyzed using NutriBase clinical data analysis software (http://www.nutribase.com/). The International Physical Activity Questionnaire (IPAQ) [24] was administered at baseline, 2 weeks and 8 weeks to characterize any changes in usual activity level during the study period that could affect study outcomes. Supplement compliance was measured by capsule counts and self-report of percentage of capsules taken. Calculated compliance was defined as the percentage of prescribed doses taken from baseline through the 8-week study period. Since differences in participants' perceptions of credibility of the treatment rationale and their expectancy could confound the findings, we administered the credibility/expectancy Questionnaire (CEQ) to participants in both groups on the first and last days of the treatment [25].

### 2.5. Statistical Analysis

All statistical analyses were performed with SPSS 16.0 (Chicago, IL). Descriptive statistics were used to characterize the sample. Nominal data were analyzed by the use of the chi-square test, whereas continuous data were analyzed by the use of Pearson's correlation analyses, independent sample *t*-tests, and one-way analysis of variance. The data are presented as mean ± SD. A significance level of 0.05 was determined *a priori*.

## 3. Results

### 3.1. Study Population

**Figure 2:**
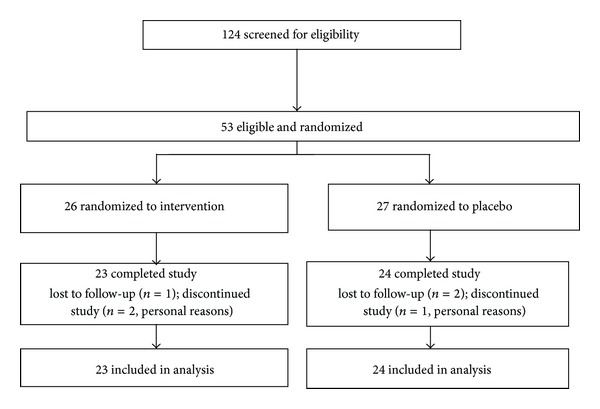
Study flow diagram.

Figure 2 depicts the screening, enrollment, and follow-up of participants in the trial. Of the 124 adults screened, a total of 53 met eligibility criteria and were enrolled in the study. Twenty-six participants were randomly assigned to the glucomannan group and twenty-seven participants to the placebo group. There were no significant differences between the two groups in rates of discontinuation. Three participants in each group were either lost to follow-up or discontinued the study for personal reasons, resulting in a final sample of 47 participants.

Baseline demographic and clinical characteristics were similar between the two groups (Table 1). Participants were predominately female (~85%), represented a mix of racial and ethnic groups, and had a mean age of 40.6 years. For the 47 participants who completed the study, the calculated compliance was 81.3% ± 4.5% in the glucomannan group and 82.7% ± 5% in the placebo group.

### 3.2. Study Outcomes

For the primary outcome, there was no significant difference in the amount of weight loss between the participants in the glucomannan group and those in the placebo group at either two weeks (−.32 ± .04 and −.11 ± .02, resp.) or eight weeks (−.40 ± .06 and −.43 ± .07, resp.) after randomization (Table 2). Results of secondary efficacy outcomes are also shown in Table 2. There were no significant differences in body composition measures, hunger/fullness, and fasting lipid and glucose levels. Belching (13.4% versus 4.1%), bloating (12.7% versus 3.7%), and stomach fullness (11.9% versus 2.4%) occurred more frequently in participants on glucomannan than those on placebo, but these symptoms were transient, lasting for only 1-2 hours after taking glucomannan on the first 1–3 study days, and did not lead to study discontinuation. Hepatic and renal safety outcomes remained normal throughout the study and did not significantly differ between the control and treatment groups. Other measures, including dietary intake, physical activity, supplement compliance, and credibility/expectancy, also did not differ significantly between the groups.

## 4. Discussion

In our study, supplementation with glucomannan did not result in significant weight loss at either 2 or 8 weeks after randomization. Also, there was no evidence of benefit of glucomannan supplementation with respect to any of the secondary outcomes. This is in contrast to several other studies that have found beneficial effects of glucomannan on body weight, body composition, and plasma lipid and glucose levels [20, 26].

Several factors may explain our study's nonsignificant findings. Unlike previous studies, we enrolled only healthy overweight and moderately obese individuals consuming self-selected diets and maintaining usual physical activity levels. As noted by Sood et al. [20], the beneficial effects of glucomannan on weight loss may be enhanced by dietary modifications, such as hypocaloric diets. Additionally, past studies have focused on obese patients, so it is possible that glucomannan may exert differential effects on these individuals compared to the overweight or moderately obese (mean BMI = 31) participants in the present study.

The lack of body composition changes may be due to the absence of an exercise intervention as part of the study design. Other trials suggest that glucomannan in conjunction with resistance and endurance exercise is necessary to promote changes in body composition, including waist and hip circumference, fat mass, and fat-free mass [26].

We also found no changes in plasma lipid or glucose concentrations. A possible explanation is that we enrolled only healthy individuals and excluded those with dyslipidemia or elevated serum glucose. Thus, floor effects may have precluded detecting any effects of glucomannan on these parameters. Another possible explanation is the lack of weight loss in our sample and its effects on these parameters [19].

Irregular eating patterns also may provide an explanation for our results. Rather than eating 3 meals, many participants reported that they “grazed” throughout the day and ate the majority of their calories in the evening, possibly circumventing our dosing schedule of 2 capsules one hour before breakfast, lunch, and dinner. Similarly, irregular eating patterns may explain the lack of difference in hunger and fullness sensations between the two groups. To effectively coordinate dosing and eating schedules, a more tailored or individualized approach should be considered.

While the dosage (3.99 g/day) of glucomannan used in our study was similar to or at the lower range of those used in other studies, a higher dosage of glucomannan should be tested in future studies. Of special interest would be whether higher doses of glucomannan might be more effective in this population. Ten grams of soluble fiber per day is considered the maximum practical dose [27].

Three limitations of this trial should be considered. First, our final sample size (*n* = 47) was relatively modest. Given the type II error that can occur with small sample sizes, this might be a possible explanation for lack of treatment effects. Second, the moderate duration of our study did not permit either long-term safety or efficacy evaluation. Glucomannan was generally well tolerated and liver enzymes and serum creatinine levels remained favorable during the 8-week study period; however, few studies have examined the long-term safety of glucomannan, and this should be a focus of future trials since extended use may impact intestinal absorption of key nutrients, particularly fat-soluble vitamins, carotenoids, and phytosterols. Similarly, glucomannan may be more beneficial over the long term when used with healthy overweight and moderately obese individuals. Third, we relied on self-report and capsule counts to monitor compliance. While participants in both groups reported a slightly greater than 80% compliance rate, it is possible that physiologic measures of compliance such as end product metabolites of glucomannan coupled with the use of electronic capsule monitoring systems would have resulted in more precise measures of compliance. In addition to phone and e-mail reminders, other technological measures to improve compliance such as text message reminders and tweets would be of interest in future studies.

## 5. Conclusions

In summary, glucomannan supplements (3.99 g daily) were well tolerated but did not promote weight loss in overweight and moderately obese individuals consuming self-selected diets and maintaining usual physical activity patterns. Other outcomes such as body composition, hunger/fullness, and lipid and glucose parameters also were not significantly altered. These results are inconsistent with the results of previous studies. Given the growing epidemic of obesity, additional studies to assess the safety and efficacy of this widely used alternative weight loss approach are needed. Future trials should evaluate glucomannan using larger numbers of participants, longer study follow-up periods, flexible dosing schedules, and higher dosages and should continue to include diverse populations of overweight and obese individuals.

## Figures and Tables

**Table 1:** Baseline demographic and clinical characteristics of study participants (*N* = 47).

Characteristic: *n* (%)	Glucomannan (*n* = 23)	Control (*n* = 24)
Gender		
Male	3 (13%)	4 (16.7%)
Female	20 (87%)	20 (83.3%)
Race/ethnic group		
White	9 (39.1%)	13 (54.2%)
Black	8 (34.8%)	6 (25.0%)
Hispanic	4 (17.4%)	5 (20.8%)
Other	2 (8.6%)	0 (0%)
Characteristic: mean (±SD)		
Age, years	35.59 (12.21)	41.59 (10.08)
Height, ft/in	5.42 (.41)	5.50 (.30)
Weight, kg	83.27 (12.32)	85.36 (12.41)
Body mass index, kg/m^2^	30.70 (2.86)	30.91 (3.28)
Waist circumference, cm	94.92 (10.25)	96.73 (11.03)
Hip circumference, cm	112.99 (8.05)	113.45 (7.41)
Fat mass, kg	32.93 (2.43)	31.91 (2.37)
Fat-free mass, kg	51.99 (3.51)	50.71 (3.61)
Total cholesterol, mg/dL	209.20 (41.82)	204.29 (31.04)
LDL cholesterol, mg/dL	135.95 (33.41)	129.76 (27.03)
HDL cholesterol, mg/dL	40.45 (8.49)	53.24 (12.50)
Triglycerides, mg/dL	123.40 (61.26)	106.90 (27.67)
Fasting glucose, mg/dL	87.05 (10.35)	86.00 (11.29)

**Table 2:** Effects of glucomannan on efficacy outcomes (*N* = 47).

Characteristic: mean	Glucomannan (*n* = 23)			Control (*n* = 24)		
	Baseline	2 weeks	8 weeks	Baseline	2 weeks	8 weeks
Weight, kg	83.75	83.43	83.36	85.4	85.3	84.97
Weight loss, kg	—	−.32	−.40	—	−.11	−.43
BMI, kg/m^2^	30.69	30.56	30.64	30.97	30.56	30.66
Waist circum., cm	95.62	94.80	94.42	97.55	96.71	97.30
Hip circum., cm	113.90	113.66	113.26	113.56	113.65	112.28
Fat mass, kg	32.93	33.09	33.31	31.91	32.21	31.91
Fat-free mass, kg	51.99	51.66	51.66	50.71	50.26	49.68
Hunger, mm	43.64	39.10	43.87	42.61	43.48	39.59
Fullness, mm	34.34	48.18	38.86	40.00	45.65	50.00
Cholesterol, mg/dL	207.00	200.06	194.50	204.60	203.13	207.33
HDL, mg/dL	47.81	45.75	48.25	54.40	52.80	52.60
LDL, mg/dL	134.00	128.94	128.12	129.47	127.47	128.73
Triglycerides, mg/dL	125.31	126.00	115.94	104.13	114.40	130.33
Glucose, mg/dL	87.38	82.79	88.06	87.93	89.33	91.00
